# Phylogeny of Anophelinae using mitochondrial protein coding genes

**DOI:** 10.1098/rsos.170758

**Published:** 2017-11-08

**Authors:** Peter G. Foster, Tatiane Marques Porangaba de Oliveira, Eduardo S. Bergo, Jan E. Conn, Denise Cristina Sant’Ana, Sandra Sayuri Nagaki, Silvio Nihei, Carlos Einicker Lamas, Christian González, Caio Cesar Moreira, Maria Anice Mureb Sallum

**Affiliations:** 1 Department of Life Sciences, Natural History Museum, London, UK; 2 Departamento de Epidemiologia, Faculdade de Saude Publica, Universidade de São Paulo, CEP 01246-904 São Paulo, SP, Brazil; 3 Superintendencia de Controle de Endemias, Secretaria de Estado da Saude de São Paulo, São Paulo, SP, Brazil; 4 Wadsworth Center, New York State Department of Health, Albany, NY, USA; 5 Department of Biomedical Sciences, School of Public Health, State University of New York-Albany, Albany, NY, USA; 6 Instituto de Biociências, Universidade de São Paulo, CEP 05508-900 São Paulo, SP, Brazil; 7 Museu de Zoologia, Universidade de São Paulo, CEP 04263-000, São Paulo, SP, Brazil; 8 Instituto de Entomología, Universidad Metropolitana de Ciencias de la Educación, Santiago, Chile

**Keywords:** *Anopheles*, mitochondrial genomes, phylogenetics

## Abstract

Malaria is a vector-borne disease that is a great burden on the poorest and most marginalized communities of the tropical and subtropical world. Approximately 41 species of Anopheline mosquitoes can effectively spread species of *Plasmodium* parasites that cause human malaria. Proposing a natural classification for the subfamily Anophelinae has been a continuous effort, addressed using both morphology and DNA sequence data. The monophyly of the genus *Anopheles*, and phylogenetic placement of the genus *Bironella*, subgenera *Kerteszia*, *Lophopodomyia* and *Stethomyia* within the subfamily Anophelinae, remain in question. To understand the classification of Anophelinae, we inferred the phylogeny of all three genera (*Anopheles*, *Bironella*, *Chagasia*) and major subgenera by analysing the amino acid sequences of the 13 protein coding genes of 150 newly sequenced mitochondrial genomes of Anophelinae and 18 newly sequenced *Culex* species as outgroup taxa, supplemented with 23 mitogenomes from GenBank. Our analyses generally place genus *Bironella* within the genus *Anopheles*, which implies that the latter as it is currently defined is not monophyletic. With some inconsistencies, *Bironella* was placed within the major clade that includes *Anopheles*, *Cellia*, *Kerteszia*, *Lophopodomyia*, *Nyssorhynchus* and *Stethomyia*, which were found to be monophyletic groups within Anophelinae. Our findings provided robust evidence for elevating the monophyletic groupings *Kerteszia*, *Lophopodomyia*, *Nyssorhynchus* and *Stethomyia* to genus level; genus *Anopheles* to include subgenera *Anopheles*, *Baimaia*, *Cellia* and *Christya*; *Anopheles parvus* to be placed into a new genus; *Nyssorhynchus* to be elevated to genus level; the genus *Nyssorhynchus* to include subgenera *Myzorhynchella* and *Nyssorhynchus*; *Anopheles atacamensis* and *Anopheles pictipennis* to be transferred from subgenus *Nyssorhynchus* to subgenus *Myzorhynchella*; and subgenus *Nyssorhynchus* to encompass the remaining species of Argyritarsis and Albimanus Sections.

## 1. Introduction

Malaria transmission is endemic in 99 countries and territories of tropical and subtropical areas of the world. Globally, approximately 3 billion people are at risk of becoming infected with *Plasmodium* parasites. The risk is variable, with some regions at high risk, whereas other areas are progressing towards elimination of malaria, or have succeeded in eliminating it [1,2]. In 2013, about 198 million cases of malaria occurred worldwide (estimates ranged from 124 to 283 million), with approximately 584 000 deaths (estimates ranged from 367 000 to 755 000), accounting for 78% of all deaths in children aged under 5 years. Even considering the uncertainties in the latest estimates of cases and deaths, malaria is a huge burden on the poorest and most marginalized communities living in endemic countries [3]. In the Americas, there were 389 390 malaria cases in 2014. Brazil accounted for 36.8% of these, followed by the Bolivarian Republic of Venezuela with 23.3% and Peru with 16.6%. These three countries together accounted for 76.7% of malaria cases in 2014; however, the highest annual parasite index (API) per 1000 people was registered in Suriname (17.4), Guyana (16.5) and Venezuela (15.3) [4]. In 2015, the Bolivarian Republic of Venezuela accounted for 30%, Brazil 24%, Peru 19% and Colombia 10% of estimated malaria cases [3]. The numbers of cases have increased because of economic conditions, mining activities and decreased vector control strategies. For instance, Venezuela reported more cases in 2014 than in any year in the previous 50 years [4].

Approximately, 41 species of the genus *Anopheles* (subfamily Anophelinae) can effectively transmit six species of the genus *Plasmodium*, *P. falciparum* (Welch), *P. vivax* Grassi & Feletti, *P. malariae* Feletti & Grassi, *P. ovale curtisi* Sutherland *et al.*, *P. ovale wallikeri* Sutherland *et al.* and *P. knowlesi* Sinton and Mulligan, from an infected to a susceptible person [5]. *Anopheles* mosquitoes also transmit the filarial parasite *Wuchereria bancrofti* Cobbold, and *Brugia malayi* Brug, as well as various arboviruses, to humans [6].

Mosquitoes belong to Culicidae, a nematocerous family of Diptera. They are subdivided into two subfamilies, Culicinae and Anophelinae. Culicinae is distributed worldwide and has 3067 species in 38 genera, including *Aedes* and *Culex*. Anophelinae has a cosmopolitan distribution with 485 formally recognized species and several unnamed members of species complexes that have not been formally described (WRBU 2016, http://wrbu.org/VecID_MQ.html). The current scheme of classification of the Anophelinae subdivides it into three genera, *Anopheles* (472 species in addition to several unnamed members of species complexes, cosmopolitan), *Bironella* (eight species, Australasian) and *Chagasia* (five species, Neotropical).

The genus *Anopheles* has eight subgenera and various informal groups as sections, series, groups and subgroups (table 1), which were defined based on morphological traits of adults, fourth-instar larvae and pupae [7,8]. Most of the sections, series, groups and subgroups are based on non-monophyletic groups (figs 4–6 in [8]). The genus *Bironella* is subdivided into three subgenera, *Bironella*, *Brugella* and *Neobironella* [9], and *Chagasia* has no subgeneric classification [10].

**Table 1.** Present scheme of internal classification of the subfamily Anophelinae (genus, subgenus, section and series), type species and number of species in each subgenus.

genus	subgenus	section	series	type species	no. species
*Anopheles*	*Anopheles*			*Anopheles maculipennis* Meigen	183
		Angusticorn	Anopheles		
			Cycloleppteron		
			Lophoscelomyia		
		Laticorn	Arribalzagia		
			Myzorhynchus		
	*Baimaia*			*Anopheles kyondawensis* Abraham	1
	*Cellia*			*Anopheles pharoensis* Theobald	224
			Cellia		
			Myzomyia		
			Neocellia		
			Neomyzomyia		
			Paramyzomyia		
			Pyretophorus		
	*Christya*			*Anopheles implexus* (Theobald)	2
	*Kerteszia*			*Anopheles boliviensis* (Theobald)	12
	*Lophopodomyia*			*Anopheles squamifemur* Antunes	6
	*Nyssorhynchus*			*Anopheles argyritarsis* Robineau Desvoidy	39
		Albimanus	Albimanus		
			Oswaldoi		
		Argyritarsis	Albitarsis		
			Argyritarsis		
		Myzorhynchella			
	*Stethomyia*			*Anopheles nimbus* Theobald	5
*Bironella*	*Bironella*			*Bironella gracilis* Theobald	2
	*Brugella*			*Bironella travestita* (Brug)	3
	*Neobironella*			*Bironella confusa* Bonne-Wepster	3
*Chagasia*				*Chagasia neivae* Cruz	5

Despite several efforts, a stable classification for the subfamily Anophelinae remains elusive. For example, relationships among the genera *Anopheles*, *Bironella* and *Chagasia* were addressed using both morphological traits [8,11,12] and DNA sequence data [13,14]. However, both morphology and molecular data failed to yield a unified consensus of the relationships among these genera. Relationships remain unresolved with contradictory hypotheses regarding the monophyly of the genus *Anopheles* and the placement of *Bironella* within the subfamily [8,13–15]. The genus *Bironella* was found either within the genus *Anopheles* as the sister group of the subgenus *Stethomyia* [11] or outside the genus *Anopheles* [14]. Recently, Harbach & Kitching [8] found *Bironella* clustered with species of *Anopheles*, without considering the possibility of the former being a subgenus of the latter. Both morphology [8,11] and DNA sequences [13,14] confirmed *Chagasia* to be a sister group to the clade composed of *Anopheles* and *Bironella* within Anophelinae. By contrast, monophyly of the genus *Anopheles* is subject to a certain degree of taxonomic instability. For instance, Krzywinski *et al.* [16] corroborated the monophyly of the genus *Anopheles* as well as of the nominal subgenus *Anopheles*, using DNA sequences of two protein-coding nuclear genes (*white* and *G6PD*), one protein-coding mitochondrial gene (*ND5*) and the D2 region of the ribosomal gene. Additionally, when the *white* gene DNA sequences were analysed separately, *Bironella* was the sister taxon of *Anopheles*. However, Sallum *et al.* [11,13], and Collucci & Sallum [17] found the genus *Anopheles* paraphyletic relative to *Bironella*. In addition, Harbach & Kitching [8,12] proposed two new subgenera (*Baimaia* and *Christya*) within *Anopheles*, but maintained *Bironella* as a valid genus even though *Bironella* and the subgenera *Stethomyia* and *Baimaia* had been placed nested within the subgenus *Anopheles*.

Currently, the genus *Anopheles* is subdivided into eight subgenera (table 1). The subgenus *Anopheles*, being cosmopolitan, has the largest geographical distribution; *Cellia* occurs in the Afrotropical, Australasian and Oriental regions; *Kerteszia*, *Lophopodomyia*, *Stethomyia* and *Nyssorhynchus* are restricted to the Neotropics, with *Anopheles* (*Nyssorhynchus*) *albimanus* reaching southern parts of the Nearctic. Little information exists concerning the distributions of subgenera *Baimaia* and *Christya*, proposed by Harbach & Kitching [8,12]. The former occurs in limited areas of Southeast Asia and was nominated to include a unique species that uses crabholes as larval habitat [18]. The subgenus *Christya* occurs in the sub-Sahara [8], and includes two sylvatic species, *Anopheles implexus* (Theobald) and *Anopheles okuensis* Brunhes, Le Goff and Geoffroy.

Phylogenetic relationships among subgenera of the genus *Anopheles* remain unresolved. Foley *et al.* [19], Sallum *et al.* [11] and Freitas *et al.* [15] found some indication that the subgenus *Anopheles* is paraphyletic. Collucci & Sallum [17] used 111 morphological characters and 36 species of *Anopheles* (*Anopheles*) with five outgroup taxa, and showed that *Anopheles* (*Anopheles*) was a monophyletic group and that *Bironella* was a sister lineage. In addition, Krzywinski *et al.* [20], based on the results of phylogenetic analysis of the DNA sequences of the *white* gene, found evidence supporting monophyly of the subgenus *Anopheles*, a sister taxa relationship of subgenera *Nyssorhynchus* and *Kerteszia*, and monophyly of a group composed of *Cellia* and *Anopheles*. Furthermore, the subgenus *Lophopodomyia* was found as sister taxon to the clade formed of *Nyssorhynchus* and *Kerteszia*, whereas the subgenus *Stethomyia* was placed outside the clade composed of other *Anopheles* subgenera. Results of a phylogenetic analysis carried out for 12 species of *Anopheles* (*Kerteszia*) confirmed the monophyly of the subgenus *Kerteszia*, and the close relationship between *Nyssorhynchus* and *Kerteszia* [21].

Foster *et al.* [22] looked at relationships within *Anopheles* (*Nyssorhynchus*), and noted that recovery of known higher-level relationships benefited from more sequence data, and by extrapolation proposed using complete mitochondrial genomes for such problems in future. The mitochondrial genome is a rich source of information and has been used in several studies [23–27]. Analysis of complete mitochondrial genomes of *Anopheles* species has provided new evidence for species complexes and a new understanding of the phylogenetic relationships among them [27–31]. Similarly, promising results have been obtained for the classification of the *Culex coronator* species complex [26]. It is remarkable that results of phylogenetic analyses, which included the mitochondrial genomes of 12 species of the lepidopteran superfamily Noctuoidea, found robust support for the monophyly of each noctuoid family [32]. It is appreciated that using complete mitochondrial genomes in phylogenetics can be problematic [24], but here the authors suggest that if gene order rearrangements, nucleotide frequency and strand bias do not vary greatly among taxa then mitochondrial genomes still have value.

Compositional bias in DNA sequences can distort the results of phylogenetic analysis, so analysis using the protein sequences derived from DNA sequences of protein-coding genes are often preferred. DNA sequences suffer from saturation more than protein sequences, partly because there are fewer character states in DNA sequences than in protein. In addition, because selection acts directly on protein sequences, but indirectly on DNA sequences, the proteins evolve more slowly than DNA. Saturation and biases such as compositional heterogeneity tend to manifest most strongly in rapidly evolving sequences, and so DNA tends to be more biased than amino acids [33–35]. In this study, we used the protein sequences of mitochondrial genomes.

In this study, family- and genus-level relationships were inferred using phylogenomic analyses of the amino acid sequences of the 13 protein coding genes of 168 newly sequenced mitochondrial genomes of Anophelinae and *Culex* species, supplemented with 23 RefSeq mitogenomes from GenBank, in order: (i) to address the monophyly of family Culicidae, and subfamilies Anophelinae and Culicinae; (ii) to define the phylogenetic position of *Bironella* and *Chagasia* within the subfamily; (iii) to establish major monophyletic groups within Anophelinae; (iv) to verify the monophyly of the subgenera *Anopheles*, *Cellia*, *Lophopodomyia*, *Kerteszia*, *Nyssorhynchus* and *Stethomyia*; and (v) to test the current classification of the subfamily Anophelinae. In this study, we provide evidence that supports an alternative hypothesis for the classification of Anophelinae based on monophyly of inferred groups drawn from mitogenomic data.

## 2. Material and methods

### 2.1. Taxon sampling

In the study, representatives of all three current genera of Anophelinae and six subgenera of the genus *Anopheles* were included in the ingroup. The species sampled for this study and the sources of specimens are listed in electronic supplementary material, table S1; the current classification of the species is in table 2. Larvae and pupae were either collected from field habitats or obtained from link-reared offspring of blood-fed females collected in the field. Both larvae and pupae were maintained in the laboratory to obtain adult males and females associated with larval and pupal exuviae. Freshly emerged mosquitoes were quickly anaesthetized with ethyl acetate, and either kept separate in minute plastic vials in silica gel or individually frozen at −80°C. One individual of *Anopheles atacamensis* was collected as an adult male in the Atacama Desert, Chile. An entire fourth-instar larva of *Bironella hollandi* was employed for the study. Species identifications were based on either adult male genitalia or fourth-instar larval morphological features. For some taxa, identification was also based on the external morphology of the eggs observed in a Jeol JSM-6460 scanning electron microscope (SEM, Jeol Ltd., Akishima, Japan) as described by Sallum *et al.* [36] and Nagaki *et al.* [37].

**Table 2.** List of species of the genus *Anopheles* employed in the present study according to current classification. Mitochondrial genome sequences of species of the subgenus *Cellia* and *Anopheles quadrimaculatus* were obtained from GenBank, and the rest were newly sequenced in this study.

subgenus	section	series	*specific epithet*
*Anopheles*	Angusticorn	Anopheles	*quadrimaculatus*
			*eiseni geometricus*
	Laticorn	Arribalzagia	*costai*
			near *costai*
			*fluminensis*
			*forattinii*
			*intermedius*
			*minor*
			*peryassui*
*Cellia*		Neomyzomyia	*cracens*
			*farauti* 4
			*hinesorum*
		Pyretophorus	*gambiae*
*Kerteszia*			*cruzii*
			*bellator*
			*homunculus*
			*leneanus*
*Lophopodomyia*			*gilesi*
			*pseudotibiamaculatus*
*Nyssorhynchus*	Albimanus	Oswaldoi	*evansae*
			*noroestensis*^*a*^
			*galvaoi*
			*konderi* A
			*konderi* B
			*konderi* C
			*oswaldoi*
			*oswaldoi* A
			*oswaldoi* SP Form
			*rangeli*
			*dunhami*
			*goeldii*
			*nuneztovari* A
			*albertoi*
			*arthuri*
			*arthuri* B
			*arthuri* C
			*arthuri* D
			*rondoni*
			*strodei*
			*striatus*
			*benarrochi*
			*triannulatus*
	Argyritarsis	Albitarsis	*albitarsis*
			*albitarsis* H
			*deaneorum*
			*marajoara*
			*oryzalimnetes*
			*braziliensis*
			near *braziliensis*
		Argyritarsis	*argyritarsis*
			*sawyeri*
			*darlingi*
			*paulistensis*^*b*^
			*lanei*
			*atacamensis*
	Myzorhynchella		*antunesi*
			*guarani*
			*lutzii*
			*lutzii* A
			*lutzii* B
			*parvus*
			*pristinus*
*Stethomyia*			*kompi*
			*nimbus*

^*a*^*Anopheles noroestensis* is currently in synonymy with *Anopheles evansae*. Specimens employed in this study are from the type locality. ^*b*^*Anopheles paulistensis* is currently in synonymy with *Anopheles darlingi*. Specimens employed in this study are from the type locality.

### 2.2. Genomic DNA isolation

DNA was extracted from each specimen individually following the animal tissue DNA extraction protocol provided by the QIAgen DNeasy^®^ Blood and Tissue Kit (QIAgen Ltd, Crawley, UK). DNA was eluted to a volume of 200 l with Buffer AE (10 mM Tris–Cl; 0.5 mM EDTA; pH 9.0) and stored at −80°C as part of the frozen entomological collection of the Faculdade de Saúde Pública, Universidade de São Paulo, Brazil. Genomic DNA extracts were used for PCR amplifications.

### 2.3. PCR amplification and sequencing

**Figure 1.**
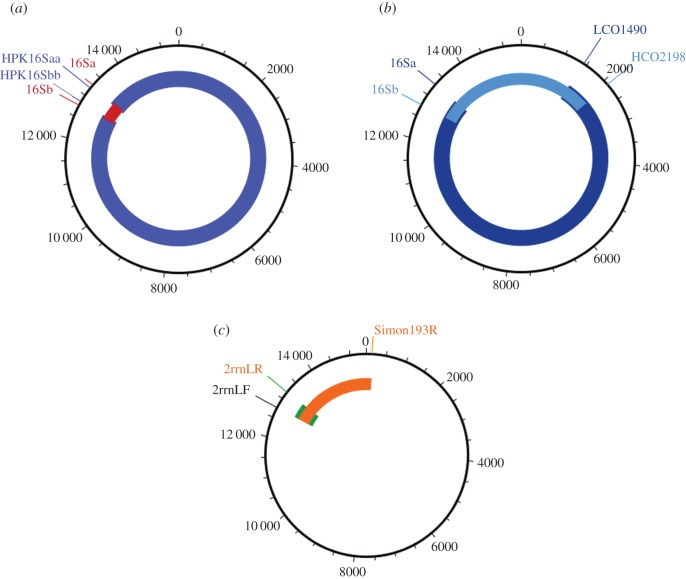
Scheme of amplifications performed in this study. Blue bars show amplified fragments sequenced by Illumina technology, while remaining colours show Sanger-sequenced fragments. (*a*,*b*) The two methods of amplification of the mitochondrial genome used in this study. In both (*a*) and (*b*), the complete mitochondrial DNA was amplified in two steps. In (*a*), fragments of about 15.058 kbp and about 655 bp were amplified and sequenced. In (*b*), the fragments about 11.857 kbp and about 4.785 kbp were amplified and sequenced in both directions using the same set of primers employed for PCR amplification. (*c*) The problematic regions after assembly of the mitochondrial genome. Some samples showed low coverage in these regions (green and orange) and then new amplifications and sequencing were carried out to complete the genome sequence. The green region in *Anopheles evansae* and *Anopheles eiseni* was amplified employing species specific primers F.

Mitochondrial genomes of Anophelinae and *Culex* species were amplified either in a single long-range PCR or two overlapping long PCR fragments using GoTaq^®^ Long PCR Master Mix (Promega, Wisconsin, USA). The PCR primers employed were conserved either across all arthropods or designed from *Anopheles* sequences (electronic supplementary material, table S3). The remaining portions of the mitochondrial genome were amplified with several primers designed for specific regions based on alignments of newly sequenced *Anopheles* DNA sequences and used for internal PCRs (electronic supplementary material, table S3). The position of the primers in the mitochondrial genome is in figure 1. The primer pairs HPK16Saa and HPK16Sbb were employed to amplify approximately 15 300 base pairs (bp), whereas the primer pairs LCO1490-16Sa amplified approximately 12 000 bp and HCO2198-16Sb approximately 4800 bp. Because PCR success varied between specimens, the amplification strategy varied according to species and specimen; for details of full amplification strategy, primers employed for varied PCRs and thermal cycling conditions, see electronic supplementary material, table S4.

The long-range PCR amplicons were purified using DNA Clean & Concentrador^*TM*^ (Zymo Research, California, USA) and quantified using the Qubit 2.0 fluorometer (LifeTechnologies, Oregon, USA). Certain regions of the mitochondrial genome were amplified by PCR using Platinum^®^ Taq DNA polymerase (Invitrogen, California, USA), and a series of primers designed for specific portions of the genome (electronic supplementary material, table S3). The PCR amplicons were purified by PEG precipitation (20% polyethylene glycol 8000/2.5 M NaCl).

### 2.4. Nextera DNA sample preparation and Illumina sequencing

Next-generation sequencing and Sanger sequencing were employed to obtain DNA sequences from 168 individuals of both Anophelinae and *Culex* species (electronic supplementary material, table S4). Long PCR products were employed to obtain barcode libraries using Nextera^®^ XT DNA Sample Preparation Kit (Illumina, Illinois, USA), and sequenced on the Illumina MiSeq platform with paired-end 250 bp chemistry.

### 2.5. Sanger DNA sequencing

For some specimens, it was problematic to obtain the entire mitochondrial DNA using Illumina sequencing technology only. Consequently, we obtained small fragments of certain portions of the mitochondrial genome to complete the circular DNA molecule. In these situations, we amplified the target DNA using primers that were developed for specific regions (electronic supplementary material, table S5). PCR products were electrophoresed in 1.0% TBE agarose gels stained with GelRed Nucleic Acid Gel Stain (Biotium Inc., Hayward, USA). Sanger sequencing reactions [38] were carried out in one direction using ABI Big Dye Terminator Kit v.3.1 (PE Applied Biosystems, Warrington, England). Sequencing reactions were purified in Sephadex G50^®^ columns (GE Healthcare), analysed on an ABI Prism 3130—Avant Genetic Analyser (Applied Biosystems, Foster City, CA, USA), and edited using Sequencher^®^ for Windows v. 5.1. Sanger DNA fragments were assembled to the mitochondrial genome obtained using Illumina sequencing technology to complete the circular molecule.

### 2.6. Sequence assembly and annotation

De novo assembly used MIRA v. 4.9 [39] and IDBA-UD v. 1.1.2 [40], aided by CAP3 [41] and visualized using Tablet [42]. MIRA was also used for assembly by mapping against very similar sequences and for mapping with extension. Blastn [43] was used to identify artefactual sequence repeats, which were excised, and for identifying overlapping ends for circularizing. Circularizing some assemblies required bridging with Sanger sequences as mentioned above.

When the sequences had been circularized, annotation began with the MITOS website http://mitos.bioinf.uni-leipzig.de [44]. Sequences were then circularly permuted so that they started with the *trnI(gat)* gene. Protein-coding genes were then checked with GeneWise (part of the Wise2 package v. 2.4 http://www.ebi.ac.uk/birney/wise2) using an HMM model (HMMER v.2, http://hmmer.org/) based on alignments of NCBI RefSeq mosquito mitochondrial translations. GenBank format files were made using tbl2asn (https://www.ncbi.nlm.nih.gov/genbank/tbl2asn2/), which were then read and manipulated using Biopython ([45], http://biopython.org/) and p4 ([46], http://p4.nhm.ac.uk). Boundaries of all genes were further checked by eye using alignments as a guide.

### 2.7. Phylogenetic analysis

The *χ*^2^-test for compositional homogeneity used p4 ([46], http://p4.nhm.ac.uk). Alignments were made using Clustal Omega [47]. Alignments were masked for reliable sites using GBlocks [48] with default settings except that parameter ‘Allowed Gap Positions’ was set to half. Duplicate sequences were removed before phylogenetic analysis, and restored with branch lengths of zero for presentation. Phylogenetic analysis used Phylobayes-MPI v. 1.5 [49], PAUP v. 4.0b10 [50], Phyml v. 20120412 [51], RAxML v. 8.1.3 [52] and IQ-Tree v. 1.5.4 [53–56]

## 3. Results

### 3.1. Newly sequenced mitogenomes, compositional heterogeneity

We sequenced 168 mosquito mitogenomes, including 148 *Anopheles*, 1 *Bironella*, 1 *Chagasia* and 18 *Culex*. The mitochondrial genomes of five species of *Anopheles* were obtained from GenBank (table 2). There were 64 *Anopheles* species, 55 of which were sequenced for the first time. Mitochondrial genomes of four species of *Anopheles* (*Kerteszia*) from the Atlantic Forest of Brazil have been described in Oliveira *et al.* [27], including *Anopheles bellator*, *An. cruzii*, *An. homunculus* and *An. laneanus*. Demari-Silva *et al.* [26] described mitochondrial genomes of four species: *Culex coronator* (two specimens), *Cu. usquatus* (one specimen), *Cu. camposi* (one specimen) and *Cu. usquatissimus* (two specimens), of the Coronator Group of *Culex* (*Culex*). Other *Culex* (*Culex*) species newly sequenced and included in the analyses as outgroup taxa were *Culex lygrus*, *Cu. nigripalpus*, *Cu. chidesteri*, *Cu. mollis*, *Cu. declarator*, *Cu. bidens*, *Cu. brami*, *Cu. dolosus* CJForm, *Cu. bilineatus* and *Cu. surinamensis*. *Culex pipiens pipiens* (NC_015079.1), *Cu. quinquefasciatus* (NC_014574.1), *Ae. notoscriptus* (NC_025473.1), *Ae. aegypti* (NC_010241.1) and *Ae. albopictus* (NC_006817.1) have been previously sequenced and were obtained from GenBank. All the mitochondrial genomes had 37 genes, and all were in the same order and on the same strand (electronic supplementary material, table S7). The 168 genomes ranged in size from 15 322 to 16 052 bp, with *Anopheles* from 15 322 to 15 739, *Culex* from 15 568 to 16 052, *Bironella* 15 772 and *Chagasia* 15 717.

**Figure 2.**
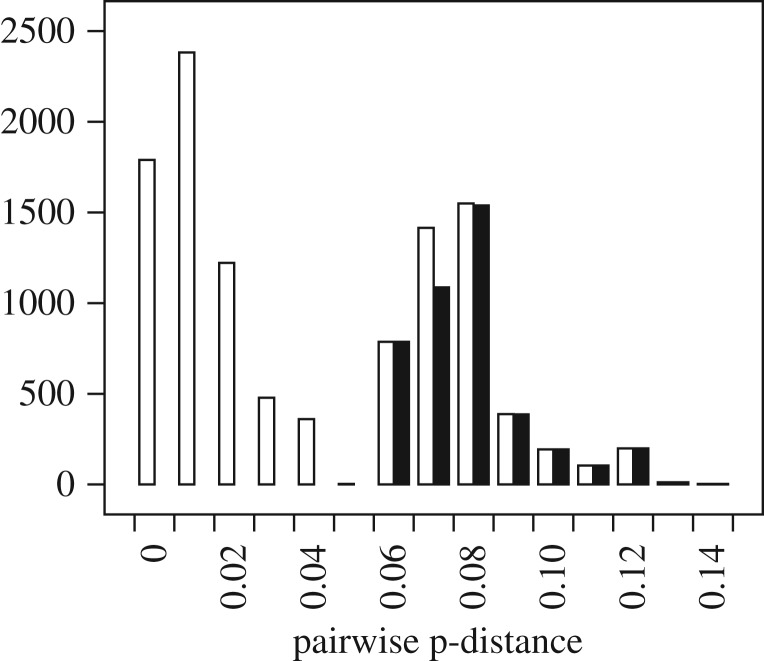
p-Distances between pairs of aligned, concatenated protein sequences, length 3735 aa. Empty bars show all p-distances except between pairs of sequences from the same species (the smallest distance in this set is 0.0005, representing two differences over the sequence pair), and filled bars show distances between taxa from different genera or subgenera (the smallest distance in this set is 0.064, representing 238 differences over the sequence pair).

The translations of the 13 protein-coding genes of the 150 newly sequenced mitogenomes of Anophelinae were similar, and after alignment GBlocks identified only one site to be masked. The translations were aligned and then concatenated to make a supermatrix of length 3735 (after masking the GBlocks site), and then compared with the corresponding DNA sequences (length 11 205). A *χ*^2^-test for compositional homogeneity did not show significant heterogeneity in the amino acid sequences (*χ*^2^=138.8; *d*.*f*.=2831; *p*=1.0) but showed substantial heterogeneity in the DNA (*χ*^2^=539.8; *d*.*f*.=447; *p*=0.0017). This test suffers from a high probability of type-II error, and so although a better test may show compositional heterogeneity in the translations, we can be sure that the DNA sequences are compositionally heterogeneous. This favours using protein sequences in subsequent phylogenetic analyses. This amino acid alignment had 772 parsimony informative sites (21% of the 3735 sites), while the corresponding DNA alignment had 4418 parsimony informative sites (39% of the 11 205 sites). Pairwise divergences between sequences in the protein alignment were examined using p-distances (figure 2), and as we were interested in relationships between genera and subgenera the protein sequences were deemed sufficiently diverged for our purpose.

### 3.2. Phylogenetic analyses

The results shown here address questions of relationships among genera in the Anophelinae and relationships among subgenera in the genus *Anopheles*. Most phylogenetic analyses were carried out using the amino acid sequences of the protein coding genes of the 168 newly sequenced mitochondrial genomes, supplemented with 23 RefSeq mitogenomes from GenBank.

Phylogenetic analysis of the Culicidae using protein sequences from mitogenomes available in GenBank with and without the new *Bironella* and *Chagasia* mitogenome sequences showed in both cases that the root of the Culicidae (mosquitoes) was between the two subfamilies (electronic supplementary material, figure S1). The rooting between the two subfamilies of the mosquitoes appears to be uncontradicted. *Bironella* was clustered within *Anopheles* (0.73 PP; electronic supplementary material, figure S1), but with *Kerteszia* sister to the clade composed of *Bironella* and *Anopheles*. The posterior probability for the branch leading to the clade composed of *Kerteszia* with *Bironella* and *Anopheles* was 1.0 (electronic supplementary material, figure S1). Examination of split supports that were not in the consensus tree shows that support for genus *Anopheles* (excluding *Bironella* and *Chagasia*) is 0.26 PP.

### 3.3. Genus-level relationships in Anophelinae rooted by Culicidae

The current generic classification of the Anophelinae includes *Chagasia*, *Bironella* and *Anopheles*, and so we would expect to see them as separate groups. However, although *Chagasia* is sister to the other groups, *Bironella* is nested within *Anopheles* (table 3; electronic supplementary material, figures S2–S7).

**Table 3.** Summary of analyses using *Culex* and *Aedes* as outgroup. Support values for *Bironella* within *Anopheles* versus monophyletic genus *Anopheles* are shown. Support for ‘*Bironella* within *Anopheles*’ is defined here as the best supported split that separates *Bironella* and some *Anopheles* taxa with the outgroup.

with RefSeq	model	*Bironella* within *Anopheles*	monophyletic *Anopheles*	electronic supplementary material, figure
+	CAT-Poisson	0.86	0.07	S2
−	CAT-Poisson	0.51	0.16	S3
+	CAT60-mtart	0.94	0.0	S4
−	CAT60-mtart	0.82	<0.01	S5
+	CAT-GTR	0.80	0.07	S6
−	CAT-GTR	0.45	0.29	S7
+	CAT-GTR	0.67	0.07	S8
−	CAT-GTR	0.52	0.19	S9

In order to see whether long branch effects were affecting the placement of *Bironella*, in analyses shown in electronic supplementary material, figures S8 and S9, several of the longest branches (except *Bironella* itself) were removed. However, the strongest support was found for *Bironella* within *Anopheles*, with little support for monophyletic *Anopheles*, suggesting that long branch effects did not affect placement of *Bironella* (table 3, last two lines).

### 3.4. Genus-level relationships in Anophelinae rooted by *Chagasia*

**Figure 3.**
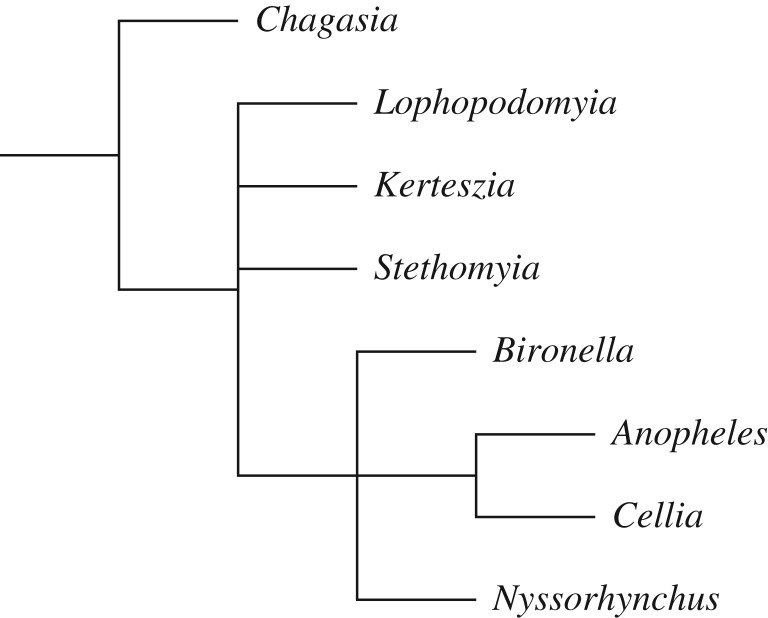
Most analyses described in this study place *Bironella* within genus *Anopheles*.

It is evident in electronic supplementary material, figures S2–S7 that *Chagasia* was the earliest branching genus in the Anophelinae, and so we will use *Chagasia* as a valid root for the rest of the Anophelinae. The series of analyses shown in electronic supplementary material, figures S10–S19 and summarized in table 4 were rooted by *Chagasia*, and used all the new *Anopheles* sequences together with *Bironella*, both with and without the nine *Anopheles* RefSeq sequences. *Culex* sequences were not used here to remove the possibility that the presence of that outgroup could distort the ingroup relationships. In many cases, there was stronger support for *Bironella* within *Anopheles* than there was for monophyletic *Anopheles* (table 4). Results of the analyses using the CAT-GTR model was an exception that showed moderate (0.72, 0.71 BPP) support for *Bironella* with *Chagasia* (electronic supplementary material, figures S10 and S11); this is counter to the CAT-GTR analyses rooted by *Culex* as shown in figures S6–S9, where support for monophyletic genus *Anopheles* was low (0.07–0.265 BPP) with this model. Placement of *Bironella* was often sister to *Anopheles* subgenera *Cellia*, *Anopheles* and *Nyssorhynchus* (figure 3) and this was equivalent to support for *Chagasia* together with *Anopheles* subgenera *Lophopodomyia*, *Kerteszia* and *Stethomyia* (LKS, not including *Bironella*), which was highest with the CAT60-MtArt model and lowest with CAT-GTR.

**Table 4.** Support for monophyletic genus *Anopheles* within Anophelinae, rooted by *Chagasia*. LKS is *Lophopodomyia*, *Kerteszia* and *Stethomyia*, subgenera of *Anopheles*.

software	model/method	RefSeq	monophyletic *Anopheles*	*Chagasia* + LKS	electronic supplementary material, figure
PB	CAT-GTR	+	0.72	0.20	S10
PB	CAT-GTR	−	0.71	0.12	S11
PB	CAT60-MtArt	+	0.01	0.905	S12
PB	CAT60-MtArt	−	0.0	0.90	S13
PB	CAT-Poisson	+	0.105	0.785	S14
PB	CAT-Poisson	−	0.045	0.84	S15
RAxML	JTT+F, RAxML-CAT^*a*^	+	0.22	0.55	S16
RAxML	JTT+F, RAxML-CAT	−	0.31	0.39	S17
PAUP*	maximum parsimony	+	0.53	0.24	S18
PAUP*	maximum parsimony	−	0.56	0.21	S19

^*a*^Prottest was used for model choice for the RAxML analysis. Prottest recommended JTT+G+F with an AICw of 1.0, and so that model was used, although for the RAxML rapid bootstrap the RAxML-CAT ASRV (among-site rate variation) was used, with only the final optimization evaluated with Gamma ASRV.

### 3.5. Anophelinae with fast sites removed

In this set of analyses fewer *Nyssorhynchus* sequences were used, and we looked at fast site removal to examine reliability of monophyletic *Anopheles*. The removal of fast sites was conducted in two ways, neither of which uses a tree:

1. Using *diversity*, that is, the number of different kinds of amino acid characters in a site [57]. It is assumed that the higher the diversity the higher will be the site rate. Data were prepared by discarding sites with a diversity higher than 3, as well as constant sites, leaving 793 of the original 1128 sites.
2. Using TIGER software [58], which identifies fast sites using compatibility. TIGER bins sites into 10 bins, and the sites in the fastest bin were removed, as well as constant sites, leaving 774 of the original 1128 sites.

The results (table 5; electronic supplementary material, figures S20–S28) of the analyses using all sites (with fewer *Nyssorhynchus*) agreed with results of previously described analyses, where the CAT-GTR model showed some small (47% and 50% in replicate analyses) support for monophyletic *Anopheles*, and the JTT analyses with RAxML and Phyml showed little (28% and 4%) support for monophyletic *Anopheles*. Using only the slow sites in the data can make the analysis more reliable, because biases in the data that may cause a lack of model fit would generally manifest in the fast sites and so their removal would be beneficial [58,59]. When this was done (table 5), support for monophyletic *Anopheles* was eroded, which seems to argue that the high support for monophyletic *Anopheles* by the CAT-GTR model was unreliable. Oddly, using JTT with RAxML and Phyml, support for monophyletic *Anopheles* increased when fast sites were removed, which appears to argue the opposite. However, there was still poor support (less than 50%) for monophyletic *Anopheles* after fast site stripping, and in spite of the ambiguity and contradictions, the tree shown in figure 3 appears to be the best summary.

**Table 5.** Summary of support values for monophyletic genus *Anopheles* using fast site stripping.

sites	*n* sites	program, model	monophyletic *Anopheles*	electronic supplementary material, figure
all	1128	Phylobayes, CAT-GTR	0.47	S20
all	1128	Phylobayes, CAT-GTR	0.50	S21
all	1128	RAxML, PROTCATJTTF	0.28	S22
all	1128	Phyml, JTT+G+F	0.04	S23
slow only, by diversity	793	Phylobayes, CAT-GTR	0.205	S24
slow only, by diversity	793	RAxML, PROTCATJTTF	0.31	S25
slow only, by diversity	793	Phyml, JTT+G+F	0.31	S26
slow only, by TIGER	774	Phylobayes, CAT-GTR	0.245	4
slow only, by TIGER	774	RAxML, PROTCATJTTF	0.48^*a*^	S27
slow only, by TIGER	774	Phyml, JTT+G+F	0.33	S28

^*a*^Note that a consensus tree made from bootstraps of the RAxML analysis of TIGER sites shows monophyletic *Anopheles* (with 48% bootstrap support) while the RAxML tree for the same analysis, which had undergone more ML rearrangements, does not.

### 3.6. Phylogenetic analysis with DNA sequences

While this study has focused on amino acid data, for comparison DNA alignments corresponding to the Anophelinae amino acid alignments including RefSeq sequences were prepared, and analysed with a partitioned ML model, and with the CAT-GTR model of PhyloBayes (electronic supplementary material, figures S29–S34). Results were broadly similar, with mostly well-supported clades of *Stethomyia*, *Lophopodomyia*, *Kerteszia*, *Anopheles*, *Cellia* and *Nyssorhynchus*. However, in contrast with the phylogenies based on translations (generally as in figure 3), support for backbone arrangements of these groups was generally poor and inconsistent using DNA. Support for *Bironella* within *Anopheles* was higher with the PhyloBayes CAT-GTR analyses, and lower for the ML analyses (table 6).

**Table 6.** Summary of support values for monophyletic genus *Anopheles* using DNA sequences.

sites	*n* sites	*n* taxa	program, model	monophyletic *Anopheles*	electronic supplementary material, figure
positions 1, 2, 3	11 202	156	IQ-Tree, partitioned	0.80	S29
positions 1, 2	7468	156	IQ-Tree, partitioned	0.91	S30
positions 1, 2, 3	11 202	156	PhyloBayes, CAT-GTR	0.76	S31
positions 1, 2	7468	156	PhyloBayes, CAT-GTR	0.40	S32
fewer taxa, positions 1, 2^*a*^	1727	61	PhyloBayes, CAT-GTR	0.25	S33
fewer taxa, positions 1, 2, slow sites^*a*^	1244	60	PhyloBayes, CAT-GTR	0.43	S34

^*a*^Constant sites removed.

### 3.7. Results summary

**Figure 4.**
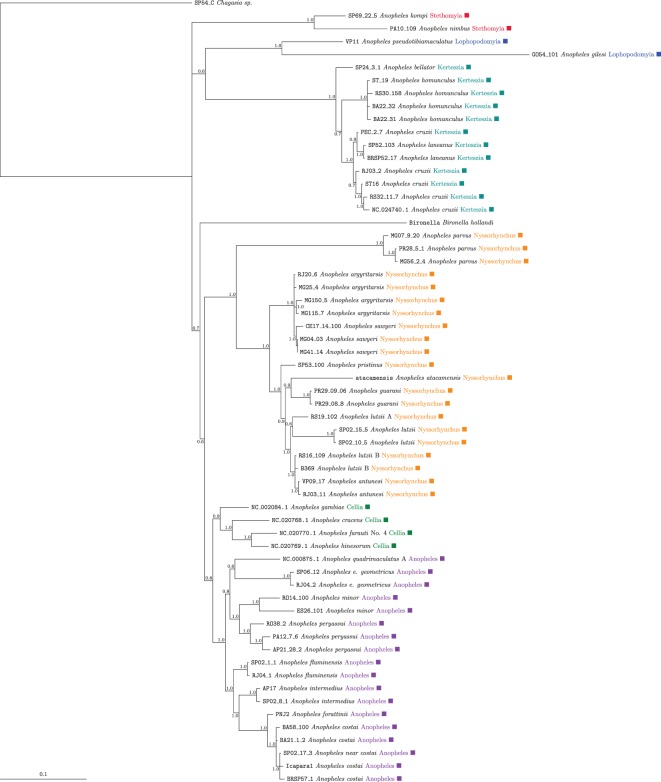
Anophelinae with reduced taxa, rooted by *Chagasia*. Mitochondrial protein sequences, slow sites only using TIGER, analysed with PhyloBayes using the CAT-GTR model.

1. With some inconsistencies, it is most likely that *Bironella* is placed within *Anopheles*.
2. Placement of *Lophopodomyia*, *Stethomyia* and the clade composed of *Cellia* with *Anopheles*, were not consistent in the analyses.
3. The current subgenera—*Stethomyia*, *Lophopodomyia*, *Kerteszia*, *Anopheles* and *Cellia*—were consistently found to be monophyletic groups.
4. The subgenus *Nyssorhynchus* was unambiguously subdivided into two strongly supported groups that were found in all analyses independent of the approach and model adopted. The *Nyssorhynchus* was subdivided into two major monophyletic groups (*BPP*=1.0) (electronic supplementary material, figures S10–S15, S20, S21, S24 and figure 4). One group was composed of *Anopheles parvus* and the second group included the remaining species of the Myzorhynchella Section plus *An. atacamensis* of the Argyritarsis Section that was recovered as sister to the group (*An. argyritarsis* plus *An. sawyeri*). Monophyly of the Argyritarsis and Albimanus Sections was not corroborated by any of the analysis and partition schemes.

## 4. Discussion

The systematic treatment of the Anophelinae has undergone extensive changes since Theobald [60], who proposed several genera based on characteristics of abdominal and thoracic scales. Subsequently, Christophers [61] named three genera based on characteristics of the male genitalia. Later, Edwards [62] and Root [63] recognized the three genera—*Anopheles*, *Myzomyia* (equivalent to *Cellia*) and *Nyssorhynchus*—as subgenera. Edwards [64] added *Stethomyia* as a subgenus of *Anopheles*, with *Kerteszia* as an informal group within the subgenus *Nyssorhynchus*. Then, Antunes [65] proposed the *Lophopodomyia* subgenus, and Komp [66] elevated *Kerteszia* to subgenus level. More recently, Harbach *et al.* [18] described the subgenus *Baimaia*, and Harbach & Kitching [8] resurrected *Christya* from synonymy with *Anopheles*. Currently, Anophelinae encompasses three genera, *Anopheles*, *Bironella* and *Chagasia*, with the genus *Anopheles* encompassing eight subgenera, of which four—*Kerteszia*, *Lophopodomyia*, *Nyssorhynchus* and *Stethomyia*—are primarily limited to the Neotropical Region [8,67] (table 1). The subdivision of the genus *Anopheles* into subgenera is based primarily on characters of the male genitalia, especially the number and placement of setae in the gonocoxite, characteristics of the ventral and dorsal claspette, aedeagus, proctiger and the ninth segment [11]. The largest subgenera in number of species are *Anopheles*, *Cellia* and *Nyssorhynchus*, and each subgenus is subdivided into several informal groups, subgroups and complexes [8,67].

Several previous studies have attempted to recover internal relationships among Anophelinae genera and among the *Anopheles* subgenera using morphology [8,11,12,21], nuclear and mitochondrial protein-coding genes [20,22], mitochondrial and ribosomal genes, among others [13,16], but the results have been unclear. That most studies were done with few taxa and few genes are among the reasons for the unsettled results, and this motivated the use of complete mitochondrial genomes and a wide taxon sampling in this study. Using mitochondrial genomes has been considered a positive advance over previously used molecular datasets for recovering interfamily relationships and for increasing support for deep nodes in phylogenies of termites [23]. However, it has also become evident that the mitochondrial genome may fail to reconstruct deep phylogenetic relationships [25,68]. Model choice can have a crucial role when using mitochondrial genomes, as in the recent study of paraneopteran orders by Li *et al.* [69]. They found big differences in substitution rates in different lineages, leading to apparent long branch attraction using site-homogeneous empirical models, which, however, was ameliorated using the site-heterogeneous CAT and CAT-GTR models as implemented in PhyloBayes. They also noted extreme saturation, and that also appeared to be accommodated well by CAT and CAT-GTR. They described the tree-heterogeneous composition of the DNA sequences, but they did not prefer use of AA sequences (which would have decreased the tree-heterogeneous composition) because using protein sequences decreased support for some groupings recovered by DNA sequences. However, a previous study using mitochondrial genomes for deep insect phylogenetics by Talavera & Vila [70] recommended using amino acid sequences to avoid long branch attraction, in addition to use of the PhyloBayes CAT model. For example it was only by using amino acid sequences with the CAT model that the Strepsiptera lineage was released from long branch attraction to the Hymenoptera, allowing it to be placed as sister to Coleoptera in agreement with current morphological and nuclear gene phylogenies. Although they had some success in avoiding long branch attraction using the CAT model with amino acid data, going deeper they were not able to recover super-order relationships reliably in insects.

In this study, we noted that the DNA sequences of our mitochondrial genomes were heterogeneous in composition, while the amino acid translations were not, as measured using a *χ*^2^-test for compositional heterogeneity (see Results, paragraph 2), and this was a major reason for us to use the amino acid sequences of the protein-coding genes. We mainly used the CAT-GTR model in PhyloBayes, but we compared this model with others. We also used long-branch taxa exclusion, fast site exclusion, and different outgroup rooting levels in order to test our results. Although there were limitations of the mitochondrial genome for inferring deep branch relationships within Anophelinae, the results of our phylogenetic analyses provided support for groups that have been previously defined based on morphological differences and similarities [60,64], and results of cladistics analyses [8,11,12], among other taxonomic studies. An analysis rooted using other nematocerous Diptera confirmed monophyly of the Culicinae family, and monophyly of the Anophelinae and Culicinae subfamilies (electronic supplementary material, figure S1).

Our analysis of relationships in Anophelinae partly contradict the current scheme of classification proposed by Harbach & Kitching [8] at the genus and subgenus levels. There is no contradiction regarding the phylogenetic systematization of the genus *Chagasia* as a monophyletic group that is sister to the clade composed of *Bironella* and *Anopheles* genera within Anophelinae. This is in agreement with other studies using either morphological characters [11,71] or different sources of DNA sequence [13,15,16,20]. However, the single representative of the genus *Bironella* included in the study, *Bironella hollandi*, was found either within the genus *Anopheles* or as its sister, depending on the analytical approach adopted and data partitioning schemes. Placement of *Bironella* nested within a more inclusive monophyletic group consisting of species of the genus *Anopheles* does not seem to be attributable to long branch attraction (table 3; electronic supplementary material, figures S8, S9, S24–S28). Consequently, the current status of *Bironella* as a genus within the Anophelinae and the monophyly of the genus *Anopheles sensu lato* are arguable. The limited sampling of some groups such as *Bironella* (one species), *Stethomyia* and *Lophopodomyia* (two species each, see below) may have contributed to the inconsistent deep relationships within Anophelinae. Thus, in order to resolve the phylogenetic position of *Bironella*, one strategy would be to use better taxon sampling; along with species from the other two *Bironella* subgenera—*Neobironella* and *Brugella*—the taxon sample should also include *Anopheles* and *Cellia* species from the Afrotropical, Indo-Malay, Australasian and Palearctic biodiversity regions. Another strategy would be to use nuclear sequences of single-copy genes and transcriptomes to overcome the problems that seem to be inherent in deep phylogenetics using mitogenomes [72–74].

Within Anophelinae, our estimated phylogenetic trees recovered relationships that are congruent with those suggested in the current classification proposed by Harbach & Kitching [8]. Species of the genus *Anopheles* consistently clustered into six major strongly supported monophyletic groups, coincident with current named subgenera: *Anopheles*, *Cellia*, *Kerteszia*, *Lophopodomyia*, *Nyssorhynchus* and *Stethomyia* (electronic supplementary material, figures S1–S28). However, in our study phylogenetic relationships among *Lophopodomyia*, *Kerteszia* and *Stethomyia* were unstable, varying depending on the method and taxon sampling. There are two major sources of instability in the classification of Anophelinae: (i) the genus *Anopheles* is probably not monophyletic because the genus *Bironella* probably lies within it (figure 3; electronic supplementary material, figure S1) and (ii) the current internal classification of the subgenus *Nyssorhynchus* is primarily based on non-monophyletic lineages (electronic supplementary material, figures S2–S28). Further, considering the presence of non-monophyletic groups within Anophelinae, we feel confident in proposing a new scheme of classification for the subfamily, mainly focused on rearrangements of subgenera of the genus *Anopheles* (table 7). Elevation of Neotropical subgenera of *Anopheles* to genus level can be justified and supported if we consider that the primary aim of any biological classification is the systematization of monophyletic supraspecific taxa, and name them formally or demonstrate their presence in nature [76,77]. Recently, Wilkerson *et al.* [78] restored Aedini classification to a generic designation that has been applied worldwide by medical entomologists. The main reasons for the decision, in the name of classification stability, were the community consensus and hall of fame criteria that are important considerations for *Aedes aegypti* and *Aedes albopictus*, among other medically important species of the genus *Aedes*. In addition, Wilkerson and colleagues also reversed the classification summarized in Reinert *et al.* [79] to allow taxonomists to accurately assign new species to a genus and to obtain additional knowledge about strongly supported monophyletic groups of species that will orient further nomenclature changes and taxon naming within Aedini.

**Table 7.** New classification proposal for the subfamily Anophelinae.

Genus *sensu* Harbach [75]*	Newly proposed genus systematization	Subgenus *sensu* Harbach [75]*	Newly proposed subgenus systematization	Type species
*Anopheles* Meigen, 1818	*Anopheles* Meigen, 1818			*Anopheles maculipennis* Meigen, 1818
		*Anopheles* Meigen, 1818	*Anopheles* Meigen, 1818	*Anopheles maculipennis* Meigen, 1818
		*Baimaia* Harbach, Rattanarithikul & Harrison	*Baimaia* Harbach, Rattanarithikul & Harrison	*Anopheles kyondawensis* Abraham, 1947
		*Christya* Theobald, 1903	*Christya* Theobald, 1903	*Anopheles implexus* (Theobald, 1903)
		*Cellia* Theobald, 1902	*Cellia* Theobald, 1902	*Cellia pharoensis* (Theobald, 1901)
	*Kerteszia* Theobald, 1905	*Kerteszia* Theobald, 1905	—	*Kerteszia boliviensis* Theobald 1905
	*Lophopodomyia* Antunes, 1937	*Lophopodomyia* Antunes, 1937	—	*Lophopodomyia squamifemur* (Antunes, 1937)
	*Nyssorhynchus* Blanchard, 1902	*Nyssorhynchus* Blanchard, 1902	*Nyssorhynchus* Blanchard, 1902	*Nyssorhynchus argyritarsis* (Robineau-Desvoidy, 1827)
			*Myzorhynchella* Theobald, 1907	*Nyssorhynchus niger* [60]; Currently, synonym of *Ny. lutzii*
	To be described		—	*Nyssorhynchus parvus* (Chagas, 1907)
	*Stethomyia* Theobald, 1902	*Stethomyia* Theobald, 1902	—	*Stethomyia nimbus* Theobald, 1902
*Bironella* Theobald, 1905	*Bironella* Theobald, 1905			*Bironella gracilis* Theobald, 1905
		*Bironella* Theobald, 1905	*Bironella* Theobald, 1905	*Bironella gracilis* Theobald, 1905
		*Brugella* Edwards, 1930	*Brugella* Edwards, 1930	*Bironella travestita* (Brug, 1928)
		*Neobioronella* Tenorio, 1977	*Neobioronella* Tenorio, 1977	*Bironella confusa* Bonne-Wepster, 1951
*Chagasia* Cruz, 1906	*Chagasia* Cruz, 1906	—	—	*Chagasia neivae* Cruz, 1906; Currently, synonym of *Chagasia fajardi*

*Reference: [75]

The classification proposed herein is supported by results of phylogenetic studies and the presence of natural groups that have been accepted by most medical entomologists. We find support for our decision when we consider the taxon naming criteria (TNC) suggested by Vences *et al.* [76]. According to these authors, taxonomists should provide a universal and stable system of classification for supraspecific taxa, and they proposed three major groups of criteria—priority, secondary and accessory, that should be considered prior to any decision about naming monophyletic supraspecific taxa and consequent nomenclature changes. The priority group includes: (i) mandatory monophyly of the taxon in an inferred species tree, (ii) clade stability derived from analyses that included various methods of tree inference, clade robustness, corroborated by a distinct set of characters and (iii) phenotypic diagnosability. The secondary and accessory groups include four criteria each, among them biogeography, manageability, hall of fame, nomenclature stability and community consensus.

In this study, we invoke the priority recommendations of Vences *et al.* as unambiguous support for elevating Neotropical *Nyssorhynchus*, *Kerteszia*, *Lophopodomyia* and *Stethomyia* subgenera to genus level. The monophyly of these taxa were always robust, independent of the analytical phylogenetic approach, taxon sampling strategy, or source of data employed for the analyses, such as morphology [11,12,17,21], nuclear and mitochondrial DNA sequence data [13,15,16] and mitogenome data as shown in this study. In addition, *Nyssorhynchus*, *Kerteszia*, *Lophopodomyia* and *Stethomyia* can be easily distinguished from the clade composed of *Anopheles* and *Cellia* based on autapomorphies of female and male genitalia or a set of morphological characters that together can be employed to distinguish these taxa from other Anophelinae genera [11,12,17,21]. The secondary TNC criteria, such as time banding, adaptive zone, hybrid viability of taxa and biogeography, cannot be used because there is not enough available information in the published literature.

The accessory TNC criteria include the manageability of a higher taxon that should contain a number of lower taxa manageable for the human mind, avoiding oversplitting and creating monotypic groups. Thus, the criteria of manageability provide extra strength to elevate *Nyssorhynchus*, *Kerteszia*, *Lophopodomyia* and *Stethomyia* monophyletic lineages to genus level. These Neotropical taxa contain few (a ‘manageable’ number) of species, and each of them can be recognized by their morphological distinctiveness from other Anophelinae genera. The genus *Anopheles* (410 species, table 1) that encompasses the species-rich subgenera *Cellia* (224 species) and *Anopheles* (183 species) is more problematic in terms of manageability and morphological diagnosability because they are not phenotypically homogeneous [11,12,17,21]. Characters of the male genitalia, whose homology has not been clearly defined, can distinguish these genera. As argued by Vences and colleagues, over-splitting a supraspecific taxon is a way to favour the principle of stability. However, this extreme situation should be avoided because it would have an undesirable impact on the evolutionary classification of organisms. The hall of fame accessory taxon naming criterion that urges taxonomists to consider the economy of change when proposing reclassification of organisms justifies our decision for not splitting the monophyletic clade composed of *Anopheles* and *Cellia* into smaller monophyletic subunits. The major reason for not splitting is that both the phylogeny and the phenotypic diagnosability are incomplete for these subgenera and thus require further study. On the other hand, the highly stable monophyly of the Neotropical *Nyssorhynchus*, *Kerteszia*, *Lophopodomyia* and *Stethomyia* subgenera justify elevating them to genus level.

Taking all the results together, we challenge the current classification of Anophelinae by proposing a revision at the genus and subgenus ranks that is consistent with our interpretation of the phylogenetic trees. Our revision preserves the six monophyletic groups that were recovered regardless of the analytical approaches adopted in the study. These are the six subgenera of *Anopheles*, the monophyly of which has been previously corroborated by morphology [8,11,12] and nuclear gene datasets [13,15,16,20,22,80]. Accordingly, the subgenera *Nyssorhynchus*, *Kerteszia*, *Lophopodomyia* and *Stethomyia* are elevated to genus rank, and the genus *Anopheles* will include the subgenera *Anopheles*, *Baimaia*, *Christya* and *Cellia* (table 7). Therefore, species assigned originally to a particular subgenus will be moved from the genus *Anopheles* to their respective newly proposed genus.

Focusing on the *Nyssorhynchus* clade, we propose adjustments in the current classification. The *Nyssorhynchus* clade is composed of two major monophyletic sister groups (electronic supplementary material, figures S1–S28). One group includes specimens of *Anopheles parvus* from the Myzorhynchella Section [81], and the second group is composed of remaining species assigned originally to the Albimanus [82], Argyritarsis [83] and Myzorhynchella [81] Sections of *Nyssorhynchus* (electronic supplementary material, figures S2–S28). Although *Anopheles parvus* had been placed in the Myzorhynchella Section on the basis of morphological similarities with other species of the section [84,85], Bourke *et al.* [86], in a phylogenetic analysis of the Myzorhynchella Section employing DNA sequences of the nuclear *white* gene, showed that *Anopheles parvus* is placed outside a more inclusive group consisting of most Myzorhynchella species. Then, Foster *et al.* [22] proposed that the species should be placed into a separate subgenus of *Anopheles* because *Anopheles parvus* is phenotypically distinguishable by unique morphological features in the egg [87] and male genitalia [84,85] in addition to the large K2P COI barcode distances compared with other *Nyssorhynchus* species. Our results here show that the Myzorhynchella Section [81] is not a monophyletic group because *Anopheles parvus* is consistently placed as a sister group to all the other *Nyssorhynchus*, separate from the other Myzorhynchella. In addition, *Anopheles atacamensis* of the Argyritarsis Section nests within the Myzorhynchella Section (see table 2, which lists the other members of the Myzorhynchella Section in the current classification). The *Myzorhynchella* were described as a genus of Anophelinae by Theobald [60] to include *Myzorhynchella nigra* Theobald. Then, the genus *Myzorhynchella* was synonomysed with *Anopheles* by Howard *et al.* [88], and redefined as a species group within the subgenus *Nyssorhynchus* by Christophers [89]. Later, Galvão [85] elevated *Myzorhynchella* to subgenus rank, which was accepted by Lane [90]. More recently, Peyton *et al.* [81] redefined Myzorhynchella as a section of the subgenus *Nyssorhynchus*. Considering that the type species of the Myzorhynchella is *Anopheles nigra* currently in synonomy with *Anopheles lutzii* Cruz, the name Myzorhynchella is preserved to be the clade that contains *Anopheles lutzii*. Elevating the *Myzorhynchella* to a subgenus of the genus *Nyssorhynchus* implies that it will encompasses *Anopheles antunesi*, *An. atacamensis*, *An. guarani*, *An. lutzii*, *An. nigritarsis*, *An. pictipennis* and *An. pristinus*. Consequently, *Anopheles parvus* will be placed into a new genus, as yet unnamed, that will be described in a further study. The second major monophyletic group of the *Nyssorhynchus* clade includes *Anopheles argyritarsis*, the type species of *Nyssorhynchus*, and thus preserves the name *Nyssorhynchus* at the genus rank. Species of the *Nyssorhynchus* genus are placed into two monophyletic groups here defined as subgenera. One subgenus contains species of the Albimanus [82] and Argyritarsis Series [83] (*sensu* [67]), except for *Anopheles atacamensis*. As *Anopheles argyritarsis* belongs to this clade, we consider it as the *Nyssorhynchus* subgenus.

## 5. Summary

With this study, we provided phylogenetic support for monophyly of Culicidae, and the subfamilies Anophelinae and Culicinae. The genus *Chagasia* is consistently the earliest branching group within Anophelinae. The phylogenetic position of *Bironella*, while not conclusive, is most likely within the current genus *Anopheles*, which implies that the latter as currently defined is not monophyletic. The subgenus *Nyssorhynchus* is sister to the clade containing *Anopheles parvus*, a species that belongs to a yet unnamed genus. *Cellia*, *Anopheles*, *Kerteszia*, *Lophopodomyia* and *Stethomyia* are monophyletic groups of the Anophelinae.

With the results of this study, we suggest modifications to the Anophelinae classification as follows:

1. Elevate the monophyletic groupings *Kerteszia*, *Lophopodomyia*, *Nyssorhynchus* and *Stethomyia* to genus level.
2. Genus *Anopheles* to include subgenera *Anopheles*, *Baimaia*, *Cellia* and *Christya*.
3. *Anopheles parvus* to be removed from *Nyssorhynchus* and to be placed into a new genus to be described in the near future.
4. Genus *Nyssorhynchus* to include two subgenera—*Myzorhynchella* and *Nyssorhynchus*.
5. *Myzorhynchella* to be elevated from a Section to subgenus rank of the newly proposed genus *Nyssorhynchus*—subgenus *Myzorhynchella*.
6. Both *Anopheles atacamensis* and *Anopheles pictipennis* to be transferred from subgenus *Nyssorhynchus* to the newly proposed subgenus *Myzorhynchella*.
7. Subgenus *Nyssorhynchus* to include species of the Argyritarsis and Albimanus Sections, except for those transferred to the *Myzorhynchella* subgenus.

We provide this alternative hypothesis for classification of Anophelinae in table 7.

## Data accessibility

DNA sequences are available at the NCBI GenBank under the following accession codes: KU551283–KU551289, MF040161–MF040165 and MF381582–MF381737. All specimen vouchers are deposited in the Universidade de São Paulo, Faculdade de Saúde Pública Collection. Data related to this paper can be found on the Dryad Digital Repository: http://dx.doi.org/10.5061/dryad.1d8th [91].
